# Integrating TiO_2_/SiO_2_ into Electrospun Carbon Nanofibers towards Superior Lithium Storage Performance

**DOI:** 10.3390/nano9010068

**Published:** 2019-01-05

**Authors:** Wenxing Liu, Tianhao Yao, Sanmu Xie, Yiyi She, Hongkang Wang

**Affiliations:** 1 State Key Laboratory for Modification of Chemical Fibers and Polymer Materials, Donghua University, Shanghai 201620, China; LiuwenxingLWX@163.com; 2 Center of Nanomaterials for Renewable Energy (CNRE), State Key Lab of Electrical Insulation and Power Equipment, School of Electrical Engineering, Xi’an Jiaotong University, Xi’an 710049, China; xjtu_yth@stu.xjtu.edu.cn (T.Y.); xiesanmu@stu.xjtu.edu.cn (S.X.); 3 Ability R&D Energy Research Centre (AERC), School of Energy and Environment, City University of Hong Kong, Hong Kong SAR 999077, China; yiyishe@cityu.edu.hk

**Keywords:** electrospinning, TiO_2_, SiO_2_, carbon nanofibers, lithium storage properties

## Abstract

In order to overcome the poor electrical conductivity of titania (TiO_2_) and silica (SiO_2_) anode materials for lithium ion batteries (LIBs), we herein report a facile preparation of integrated titania–silica–carbon (TSC) nanofibers via electrospinning and subsequent heat-treatment. Both titania and silica are successfully embedded into the conductive N-doped carbon nanofibers, and they synergistically reinforce the overall strength of the TSC nanofibers after annealing (Note that titania–carbon or silica–carbon nanofibers cannot be obtained under the same condition). When applied as an anode for LIBs, the TSC nanofiber electrode shows superior cycle stability (502 mAh/g at 100 mA/g after 300 cycles) and high rate capability (572, 518, 421, 334, and 232 mAh/g each after 10 cycles at 100, 200, 500, 1000 and 2000 mA/g, respectively). Our results demonstrate that integration of titania/silica into N-doped carbon nanofibers greatly enhances the electrode conductivity and the overall structural stability of the TSC nanofibers upon repeated lithiation/delithiation cycling.

## 1. Introduction

Rechargeable lithium ion batteries (LIBs) have been widely applied in portable electronics owing to their outstanding advantages such as long lifespan, little memory effect, high energy density, high working voltage, low self-discharge, and eco-friendliness [1,2,3,4]. With increasing demand for higher energy/power densities, various new alternative anode materials have been widely studied, in order to replace the current commercial graphite based anodes which have a lower theoretical capacity of 372 mAh/g. Titanium dioxide (TiO_2_) as a typical intercalation-type anode material has received considerable attention owing to its long cycle life, low-cost, low volume variation (<4%) and environmental friendliness [5,6,7,8,9]. Especially for the nano-sized TiO_2_, the intercalation–deintercalation reaction TiO_2_ + xLi^+^ + xe^−^ ↔ Li_x_TiO_2_ (x ≤ 1) can provide a high theoretical specific capacity of 335 mAh/g [10,11,12]. However, the TiO_2_ anode suffers from its low intrinsic electrical conductivity (10^−12^ S·cm^−1^) and thus resulting poor lithium storage performance.

Silica (SiO_2_) is an insulating oxide and generally electrochemically inactive in bulk, but nanosized SiO_2_ could react with Li in 0~1.0 V (vs. Li/Li^+^), which theoretically possesses a high specific capacity of 1965 mAh/g (albeit controversial) [13,14,15,16,17,18,19,20,21]. For example, Guo et al. reported that the as-prepared nano-SiO_2_/hard carbon composite can deliver a discharge capacity of 630 mAh/g. Yao et al. demonstrated that a carbon-coated SiO_2_ displayed a reversible capacity of 500 mAh/g at 50 mA/g [19]. Sasidharan et al. reported the fabrication of hollow silica nanospheres, which exhibited superior cycle stability with a discharge capacity of 336 mAh/g after 500 cycles at 1C [22]. Notably, Yan et al. successfully developed hollow porous SiO_2_ nano-cubes which showed a high reversible capacity of 919 mAh/g when cycled at 100 mA/g after 30 cycles [21]. Therefore, delicate structure design of silica-based anodes with the aim to buffer the volume changes and increase the electrical conductivity can largely enhance the lithium storage performance. Besides, silica is cheap and abundant on the Earth, which holds great potentials in LIB applications [23,24,25].

Encapsulating electrode materials into carbon-based matrices has been an efficient strategy to enhance the lithium storage performance [26,27,28,29,30]. Electrospinning is one of the promising methods to prepare carbon fibers and various exotic anode materials can be easily embedded, thus the electrical conductivity can be improved and the volume expansion can be accommodated [4,17,31]. We have demonstrated that incorporation of silica into carbon nanofibers can not only enhance the structure stability of SnSb/C and Sb/C nanofibers, but also their electrochemical properties [24,25]. Herein, we further report the facile fabrication of novel integrated titania–silica–carbon nanofibers through electrospinning and subsequent annealing, and both titania and silica have been fully incorporated into the carbon nanofibers, which showed superior lithium storage performance when used as anode materials.

## 2. Experimental Section

### 2.1. Materials Fabrication

All the chemicals were adopted as received without any further treatment. Electrospinning method was used to prepare the integrated titania–silica–carbon (TSC) nanofibers. In a typical synthesis, 0.5 mL tetraethyl orthosilicate (TEOS, Si(OC_2_H_5_)_4_, J&K) and 1.0 mL tetrabutyl titanate (TBT, C_16_H_36_O_4_Ti, J&K) were dissolved in a mixed solvent of *N*,*N*-dimethylformamide (DMF, C_3_H_7_NO, J&K, 4 mL) and isopropanol (C_3_H_8_O, J&K, 2 mL) under vigorous stirring at room temperature. To inhibit the hydrolysis of TBT, 0.1 mL HCl (37 wt.%) were added into the above mixture. When the solution became clear, 0.72 g polyvinylpyrrolidone (PVP, (C_6_H_9_NO)_n_, M_W_ = 1,300,000, J&K) were introduced under magnetically stirring until the complete dissolution. The precursor solution was then transferred into a plastic syringe, and the distance between the needle and the fiber collector was kept at around 15 cm. For the electrospinning, the voltage was set at 18 kV, and the flow rate was 0.5 mL/h, using an aluminum foil as the fiber collector. For the subsequent annealing to obtain the final TSC product, the as-spun precursor nanofibers were first dried at 80 °C and then at 250 °C for 1 h in air, which were finally annealed at 800 °C for 2 h under Ar at a rate of 5 °C/min. Note that titania–carbon or silica–carbon nanofibers can not be obtained under the same condition (Figure S1).

### 2.2. Structural Characterization

Powder X-ray diffraction (XRD, Bruker D2 PHASER) was applied to investigate the phase structures of the products by using Cu Kα radiation (λ = 1.5418 Å) at a voltage of 30 kV and a current of 10 mA. Scanning electron microscope (SEM, FEI Quanta 250F) and transmission electron microscope (TEM, JEM-F200) were used to examine the morphological structures of the products. Elemental distribution analysis was performed using high-angle annular dark field scanning transmission electron microscopy (HAADF-STEM) equipped with energy-dispersive X-ray spectroscopy (EDS). X-ray photoelectron spectroscopy (XPS, Thermo Fisher ESCALAB 250Xi+) was adopted to study the compositions as well as the chemical states of the products. Thermogravimetric analysis (TGA) was performed on a METTLER TOLEDO TGA/DSC thermal analyzer, and Raman spectroscopy was performed on Renishaw Raman RE01 scope using Ar excitation laser (514 nm). The specific surface area and the pore structure characteristics were revealed by the Brunauere–Emmette–Teller (BET) method, using a Quantachrome Surface Area Analyzer (Autosorb iQ-MP), and the N_2_ sorption isotherms were acquired at 77 K.

### 2.3. Electrochemical Measurements

The lithium storage properties of the as-prepared TSC nanofibers were evaluated using 2025 coin-type cells, which were assembled in an Ar-filled glovebox with both H_2_O and O_2_ contents less than 1.0 ppm. For the fabrication of the working electrode, slurry containing active materials (TSC), conductive agent (Ketjenblack, Carbon ECP-600JD) and binder (polyacrylic acid, PAA, Mw = 100,000, Sigma) with a weight ratio of 7:2:1 was first prepared using distilled water as solvent, and then the slurry was cast onto a copper foil via a doctor blade method, followed by drying in a vacuum oven at 120 °C overnight. Lithium foil was used as the counter and reference electrode, and the microporous membrane (Celgard 2400) was applied as separator. The adopted electrolyte was prepared by dissolving 1 M LiPF_6_ in the dimethyl carbonate/ethylene carbonate (1:1 *v*/*v*). Galvanostatic discharge-charge profiles were obtained using a NEWARE battery test system (Neware Technology Co., Ltd., Shenzhen, China) in 0.01–3.0 V (vs. Li^+^/Li) at 25 °C. Cyclic voltammetry (CV) measurements and electrochemical impedance spectroscopy (EIS) analysis were both conducted on an Autolab electrochemical workstation (PGSTAT 302N). The CV curves was obtained in 0.01–3.0 V at different scan rates 0.1–1.0 mV/s, and EIS was performed with a voltage amplitude of 10 mV in the frequency range of 10^5^–0.01 Hz. The specific capacity was based on the active materials of TSC nanofibers, and the loading amount in each working electrodes was approximately 0.8–1.0 mg cm^−2^.

## 3. Results and Discussion

**Figure 1:**
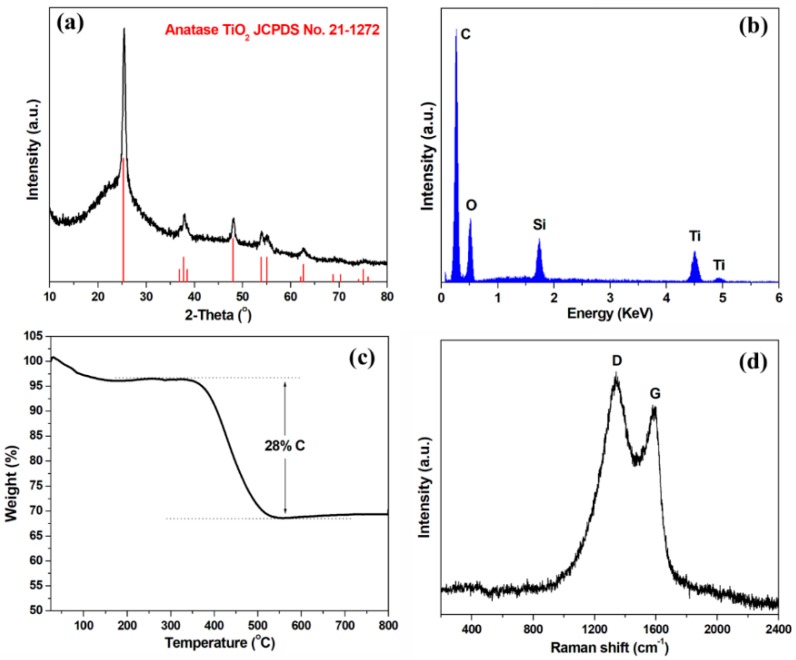
(**a**) XRD pattern, (**b**) EDS spectrum, (**c**) TGA curve and (**d**) Raman spectrum of TSC nanofibers.

Figure 1a displays the XRD pattern of the TSC nanofibers, in which all the apparent peaks can be indexed to the anatase TiO_2_ (JCPDS No. 21-1272). No peaks corresponding silica can be observed, indicating its amorphous nature in the hybrid. The broad peak at ~25° can be related to the graphite carbon. Even though no XRD peaks for silica can be observed, the EDS spectrum clearly reveals the presence of Si element as well as the Ti, O and C elements (Figure 1b). The Ti/Si atomic ratio is approximately 1.30 (Figure S2), which is consistent with the theoretical value. In order to determine the relative contents of titania, silica and carbon, thermogravimetric analysis (TGA) was performed. As shown in Figure 1c, the carbon content in the TSC nanofibers is ~28 wt.%, thus the contents of titania and silica can be estimated to be 45.5 and 26.5 wt.%, respectively. Raman spectroscopy was employed to study the graphitic crystallization and the molecular vibrations in the TSC nanofibers. As shown in Figure 1d, two prominent peaks are observed at ~1348 and ~1596 cm^−1^, which correspond to the D-band and G-band of graphite [32], respectively. The I_D_/I_G_ intensity ratio for TSC is 1.1, indicating a high defect degree of the carbon nanofibers. Besides, no apparent peaks for TiO_2_ and SiO_2_ can be observed, which may be owing to their full incorporation into the carbon matrix.

**Figure 2:**
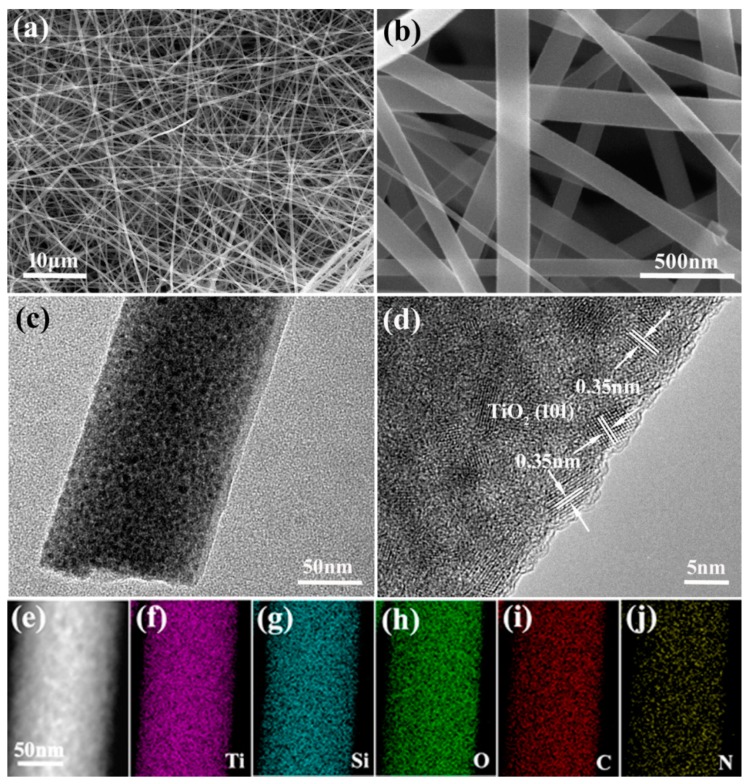
(**a**,**b**) SEM images, (**c**) TEM and (**d**) HRTEM images of TSC nanofibers. (**e**) HAADF-STEM image of a single TSC nanofiber with corresponding EDS maps of (**f**) Ti, (**g**) Si, (**h**) O, (**i**) C and (**j**) N elements.

Figure 2a shows the SEM image of the TSC nanofibers, which have lengths of tens of micrometers, and the fibers show smooth surface with diameters of 80~160 nm (Figure 2b). TEM and HRTEM images further reveal the inner microstructures of the TSC nanofibers, as shown in Figure 2c,d. The ultrafine nanoparticles with sizes of 2–6 nm are fully encapsulated within the fibers, and the lattice fringes with d spacing of 0.35 nm can be indexed to the (101) planes of anatase TiO_2_ (Figure 2d), which is consistent with the XRD result. Furthermore, HAADF-STEM image of a single TSC nanofiber is shown in Figure 2e, where EDS maps were taken in order to reveal the element distribution. As shown in Figure 2f–j, the Ti, Si, O, C and N elements are uniformly distributed within the fiber, indicating the full encapsulation of TiO_2_ and SiO_2_ into the carbon nanofibers. The specific surface area and the pore feature were revealed by the N_2_ sorption isotherms (Figure S3), which display a small surface area of 7.6 m^2^/g and a small pore volume of 0.07 cc/g, indicating the solid structure of the TSC nanofibers. These results are consistent with the TEM observation, in which both titania and silica are closely embedded within the carbon matrix, and no apparent pore can be observed within the integrated TSC nanofibers.

**Figure 3:**
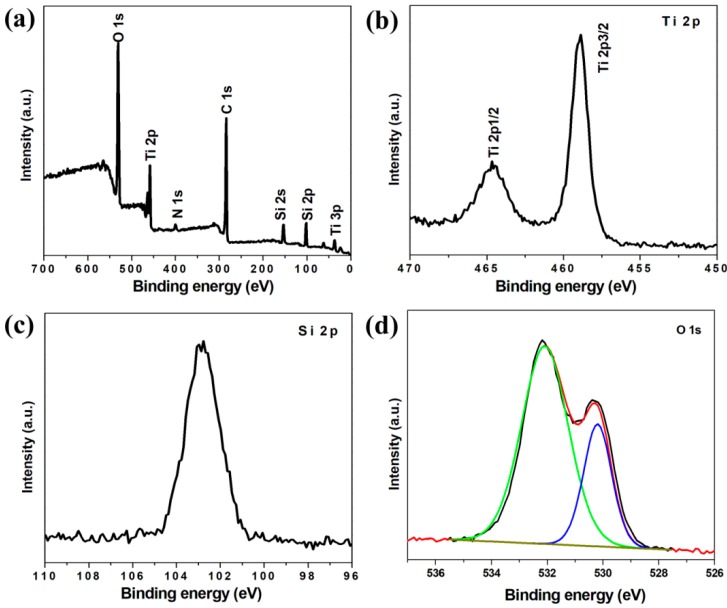
(**a**) Survey XPS spectrum of TSC nanofibers and the corresponding high-resolution (**b**) Ti 2p, (**c**) Si 2p and (**d**) O 1s XPS spectra.

X-ray photoelectron spectroscopy (XPS) was performed to determine the chemical composition and the elemental states of the TSC nanofibers. Figure 3a shows the survey XPS spectrum, in which we can clearly see the presence of the Ti, Si, O, N and C elements. In the Ti 2p XPS spectrum (Figure 3b), the doublet peaks at 464.6 and 458.9 eV can be indexed to Ti 2p1/2 and Ti 2p3/2 with the highest oxidation state of Ti(IV) [33], confirming the formation of TiO_2_ in the TSC nanofibers. As shown in the Si 2p XPS spectrum (Figure 3c), the broad peak centered at around 103.0 eV reveals the oxidation state of Si(IV) [24]. In Figure 3d, the O 1s XPS spectrum shows two well-defined peaks located at around 532.0 and 530.0 eV, which are corresponding to the Si-O and Ti-O, respectively [16].

**Figure 4:**
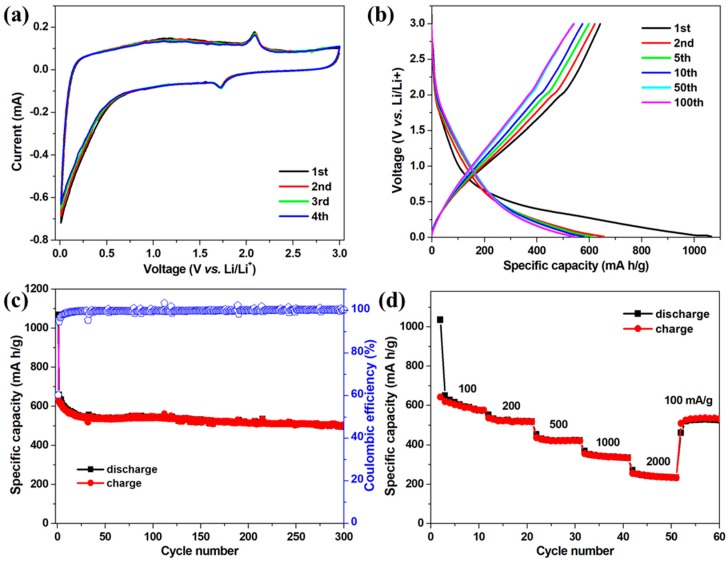
(**a**) CV curves at a scan rate of 0.1 mV/s; (**b**) galvanostatic discharge-charge profiles at 100 mA/g; (**c**) cycle performance at 100 mA/g; and (**d**) rate performance of TSC nanofibers at different current densities.

The electrochemical properties of the TSC nanofibers as LIB anode materials were examined in coin-type cells using lithium foil as counter and reference electrode. Figure 4a shows the CV curves of the TSC nanofiber electrode. A pair of cathodic/anodic peaks can be clearly observed at 1.7/2.1 V, corresponding to the lithium insertion/extraction in the TiO_2_ lattice (TiO_2_ + xLi^+^ + xe^−^ ⇌ Li_x_TiO_2_) [34,35,36,37]. The sharp cathodic peak near 0.1 V can be associated with the lithium storage in the carbon and silica components [24,25]. Correspondingly, less apparent but broad peaks in the anodic scans can be observed, which is typical for the carbon and silica anodes. More importantly, it is worth mentioning that the CV curves are well overlapped in the first four cycles, indicating the highly reversible electrochemical reactions in the TSC electrode. Figure 4b shows the galvanostatic discharge-charge profiles of the TSC electrode at 100 mA/g. A minor plateau at ~1.7 V in the discharge process and the corresponding plateau in the charge process can still be observed, which relates to the lithium insertion/extraction in TiO_2_. The capacity below 0.5 V mainly originates from lithium insertion into the graphitic carbon layers and silica anode, while the capacity above 0.5 V could be ascribed to the Faradaic capacitance on the surface or on the edge sites [38]. The initial discharge/charge capacities for TSC electrode are 1066.4/642.6 mAh/g, giving an initial Coulombic efficiency of ~60%. The capacity loss can be due to the formation of solid electrolyte interphase (SEI) film and the irreversible reaction between silica and lithium by forming lithium silicates in the first cycle [16,24]. Figure 4c shows the cycle performance of TSC electrodes, which deliver high reversible capacities of 502 mAh/g after 300 cycles at 100 mA/g, respectively, with a capacity retention of ~80%. Figure 4d shows the rate performance of TSC electrode, which delivers reversible capacities of 572, 518, 421, 334, and 232 mAh/g each after 10 cycles at 100, 200, 500, 1000 and 2000 mA/g, respectively. When recycled at 100 mA/g, a high reversible capacity of 526 mA/h still maintains after 10 cycles, in which the contribution of silica can be calculated as ~1017 mAh/g (372 mAh/g × 0.28 (for carbon) + 335 mAh/g × 0.455 (for TiO_2_) + 1017 mAh/g × 0.265 (for SiO_2_) ≈ 526 mAh/g).

**Figure 5:**
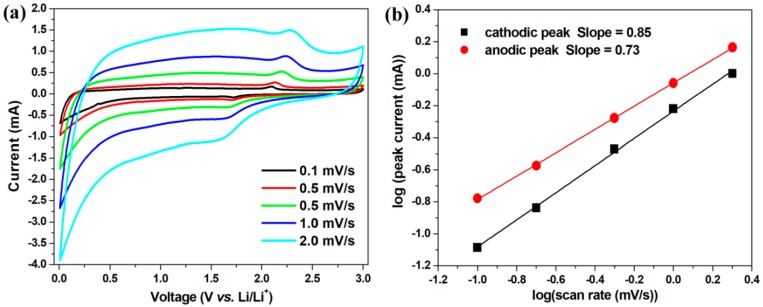
(**a**) CV curves of TSC electrode at different scan rates and (**b**) the corresponding log-log plots of the cathodic/anodic peak currents versus the scan rates.

It is well known that the lithium storage mechanism of TiO_2_ is based on the insertion/extraction of Li ion in TiO_2_ crystal. However, nanosized particles can provide additional Li-storage capacity via surface reaction [7,39]. To further explore the charge storage mechanism of the TSC hybrid, CV tests were performed at different scan rates (Figure 5a). For a redox reaction, the peak current *i* and the scan rate *v* obeys the power-law equation $i=av^{b}$ [38,40,41,42], where *a* and *b* are fitting parameters. The slope of the fitting line is the *b* value in the log(i)-log(v) plot, which reflects different charge storage mechanism. Generally, *b* = 1 indicates a capacitive behavior, while *b* = 0.5 implies a diffusion-limited process. As shown in Figure 5b, the b values for the fitting lines arising from the cathodic peaks in 1.5–2.0 V and the anodic peaks in 2.0–2.5 V are 0.85 and 0.73, respectively. Both b values in the range of 0.5–1.0 indicate that the electrochemical mechanism is both diffusion-limited and capacitive. The fast Faradaic pseudocapacitive process happens in the surface layers of nanosized particles, which not only contributes to additional Li-storage capacity, but also a high rate capability.

**Figure 6:**
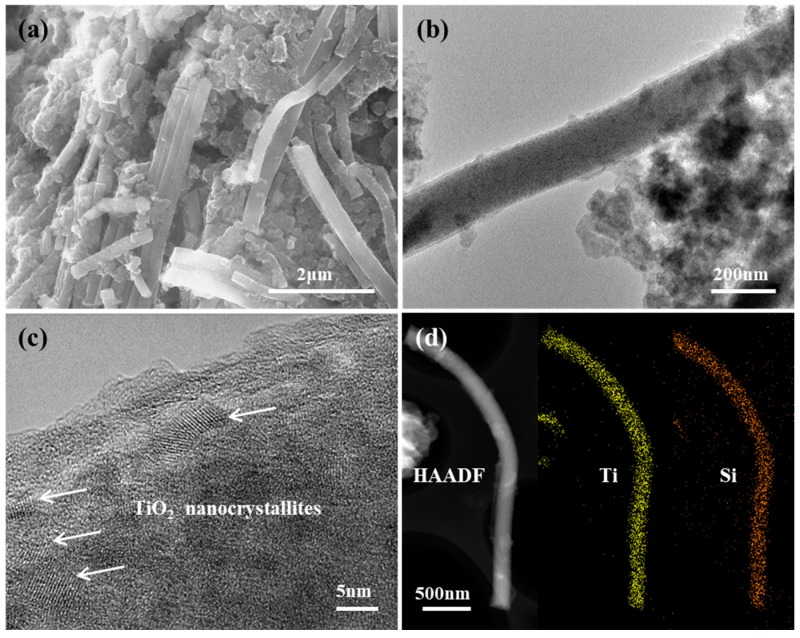
(**a**) SEM, (**b**) TEM, (**c**) HRTEM images of the TSC nanofiber electrode after 300 cycles at 100 mA/g. (**d**) HAADF-SEM image with corresponding EDS maps of Ti and Si elements in a single TSC nanofiber after cycling.

Ex situ SEM and TEM analyses were further performed to reveal the structure evolution after cycling. As shown in Figure 6a, the fibrous morphology was well preserved after 300 cycles at 100 mA/g, indicating the superior structural stability. TEM image also reveals the maintenance of fiber structure (Figure 6b), and HRTEM image still shows clear lattice fringes with d spacing of 0.35 nm (Figure 6c), which can be indexed to the (101) plane of anatase TiO_2_, indicating the TiO_2_ nanoparticles are still well embedded within the carbon nanofibers. Moreover, the HAADF-STEM image and the corresponding EDS maps of Ti and Si elements further confirm the structural stability of the TSC nanofibers, in which no phase aggregation can be observed (Figure 6d). Electrochemical impedance spectroscopy (EIS) was also carried out to study the charge transfer resistance of the electrodes. Figure S4 shows the Nyquist plots of the TSC electrodes at different states, in which the semicircle diameter represents the charge transfer resistance (R_ct_). The fresh TSC electrode exhibits a small R_ct_ of around 80 Ω, which resolves into two peaks corresponding to the SEI resistance and the charge transfer, respectively, but still remains small even after long-term cycling, indicating the fast charge transfer rate. In addition, the inclined lines at the low frequency are quite steep, indicating the fast lithium ion diffusion processes [32].

## 4. Conclusions

In summary, integrated titania–silica–carbon (TSC) nanofibers have been successfully prepared via a facile electrospinning method. The titania nanoparticles with size of 2–6 nm were fully encapsulated and uniformly distributed within the silica-carbon matrices, while the in situ generated silica facilitated to maintain the fibrous structure of the TSC hybrid during the calcination. When used as the anode materials for lithium ion batteries, the TSC hybrid demonstrated superior lithium storage properties including high cycle stability and rate capability, delivering high discharge capacities of 502 mAh/g at 100 mA/g after 300 cycles, respectively. The silica component with a content of 26.5 wt.% contributed a specific capacity of ~1017 mAh/g. The superior electrochemical performance was attributed to the novel structure characteristics. The one-dimensional carbon nanofibers not only greatly enhanced the electrode conductivity, which also fully encapsulated the titania and silica components, thus facilitating to maintain the overall structure integrity. More importantly, the introduction of silica into the electrospun carbon nanofibers not only reinforced the overall structure stability, but also brought additional lithium storage capacity, and the concept can be generally applied for the design of high-performance electrochemical energy storage electrodes.

## Supplementary Materials

The following are available online at http://www.mdpi.com/2079-4991/9/1/68/s1. Figure S1: SEM images of (a) the TBT/PVP nanofibers (without TEOS) and the counterpart after annealing, (c) SEM and (d and inset) TEM images of the annealed TEOS-PVP fibers under the same condition, Figure S2: EDS spectra of the TEOS/TBT/PVP precursor nanofibers, TSC and TS (obtained after TGA analysis) nanofibers, Figure S3: N_2_ adsorption-desorption isotherms of the TSC nanofibers, with inset showing the corresponding pore size distribution, Figure S4: Nyquist plots of the fresh TSC electrode.
