# Supplementary material for: Integrating TiO2/SiO2 into Electrospun Carbon Nanofibers towards Superior Lithium Storage Performance
Source: Nanomaterials (Basel). 2019 Jan 5;9(1):68. doi: 10.3390/nano9010068 (PMC6359262; doi:10.3390/nano9010068)
Supplement: Supplementary file 1 [file nanomaterials-09-00068-s001.pdf]

Supporting Information

Integrating TiO_2/SiO_2 into Electrospun Carbon Nanofibers towards Superior Lithium Storage Performance

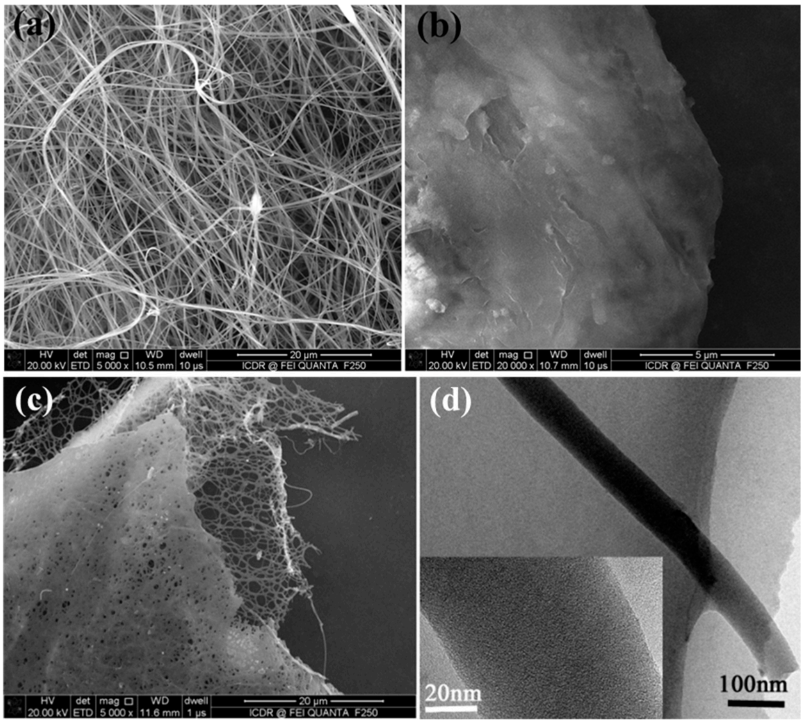

Figure 1. SEM images of (a) the TBT/PVP nanofibers (without TEOS) and the counterpart after annealing. (c) SEM and (d and inset) TEM images of the annealed TEOS-PVP fibers under the same condition. These results reveal that the TEOS derived silica and TBT-derived titania can simultaneously facilitate to maintain the fibrous morphology.

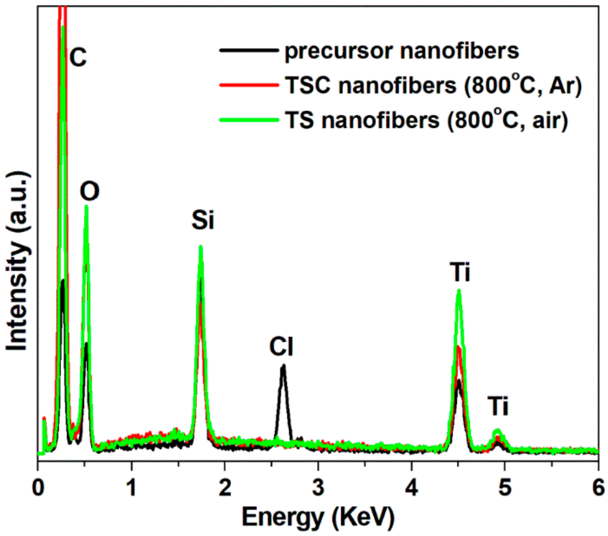

Figure S2. EDS spectra of the TEOS/TBT/PVP precursor nanofibers, TSC and TS (obtained after TGA analysis) nanofibers, which revealed the Ti/Si atomic ratio was almost constant at ~1.3.

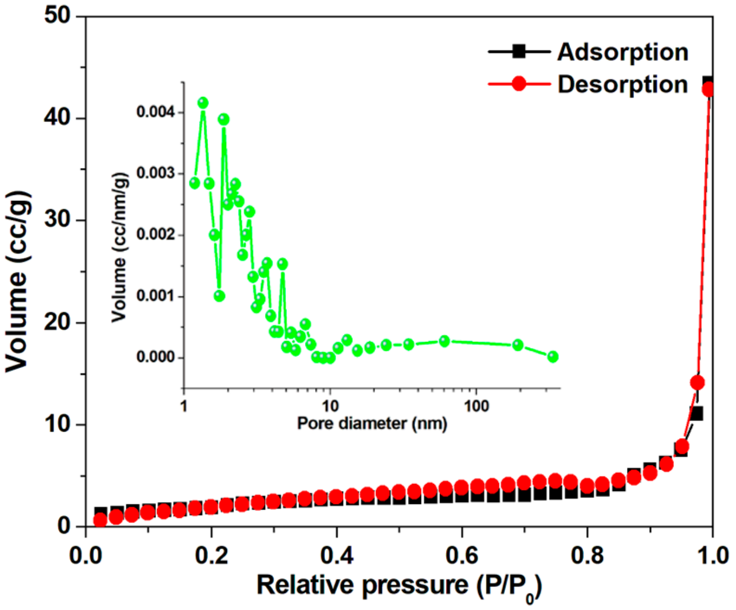

Figure S3. N_2 adsorption-desorption isotherms of the TSC nanofibers, with inset showing the corresponding pore size distribution.

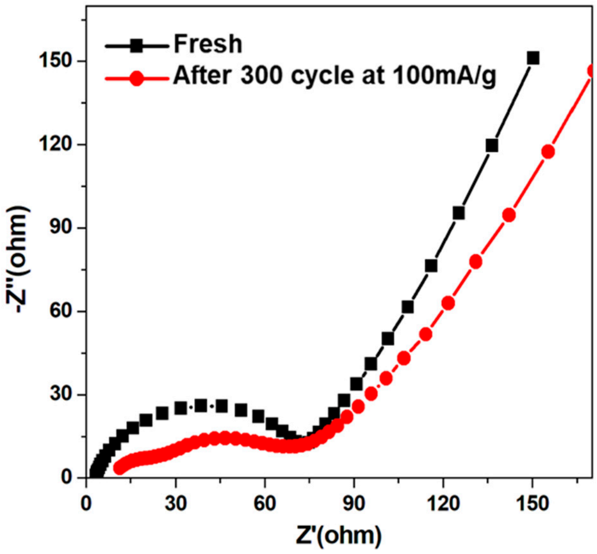

Figure S4. Nyquist plots of the fresh TSC electrode at OPV of 2.85V and its counterpart after cycling at 1000 mA/g at OPV of 2.62 V (OPV: open circuit voltage).
